# Chronological brain lesions after SARS-CoV-2 infection in hACE2-transgenic mice

**DOI:** 10.1177/03009858211066841

**Published:** 2021-12-27

**Authors:** Enric Vidal, Carlos López-Figueroa, Jordi Rodon, Mónica Pérez, Marco Brustolin, Guillermo Cantero, Víctor Guallar, Nuria Izquierdo-Useros, Jorge Carrillo, Julià Blanco, Bonaventura Clotet, Júlia Vergara-Alert, Joaquim Segalés

**Affiliations:** 1 IRTA, Centre de Recerca en Sanitat Animal (CReSA, IRTA-UAB), Campus UAB, Bellaterra, Barcelona, Catalonia, Spain; 2 Barcelona Supercomputing Center (BSC), Jordi Girona, Barcelona, Spain; 3 Catalan Institution for Research and Advanced Studies (ICREA), Barcelona, Spain; 4 IrsiCaixa AIDS Research Institute, Badalona, Spain; 5 Germans Trias i Pujol Research Institute (IGTP), Can Ruti Campus, Badalona, Spain; 6 University of Vic-Central University of Catalonia (UVic-UCC), Vic, Spain; 7 Departament de Sanitat i Anatomia Animals, Facultat de Veterinària de la UAB, Campus UAB, Bellaterra, Barcelona, Catalonia, Spain; 8 UAB, Centre de Recerca en Sanitat Animal (CReSA, IRTA-UAB), Campus UAB, Bellaterra, Barcelona, Catalonia, Spain

**Keywords:** SARS-CoV-2, Coronavirus Disease 2019, neuropathology, hACE2-transgenic mice, brain, meningoencephalitis, animal model

## Abstract

Severe acute respiratory syndrome coronavirus 2 (SARS-CoV-2) causes respiratory disease, but it can also affect other organs including the central nervous system. Several animal models have been developed to address different key questions related to Coronavirus Disease 2019 (COVID-19). Wild-type mice are minimally susceptible to certain SARS-CoV-2 lineages (beta and gamma variants), whereas hACE2-transgenic mice succumb to SARS-CoV-2 and develop a fatal neurological disease. In this article, we aimed to chronologically characterize SARS-CoV-2 neuroinvasion and neuropathology. Necropsies were performed at different time points, and the brain and olfactory mucosa were processed for histopathological analysis. SARS-CoV-2 virological assays including immunohistochemistry were performed along with a panel of antibodies to assess neuroinflammation. At 6 to 7 days post inoculation (dpi), brain lesions were characterized by nonsuppurative meningoencephalitis and diffuse astrogliosis and microgliosis. Vasculitis and thrombosis were also present and associated with occasional microhemorrhages and spongiosis. Moreover, there was vacuolar degeneration of virus-infected neurons. At 2 dpi, SARS-CoV-2 immunolabeling was only found in the olfactory mucosa, but at 4 dpi intraneuronal virus immunolabeling had already reached most of the brain areas. Maximal distribution of the virus was observed throughout the brain at 6 to 7 dpi except for the cerebellum, which was mostly spared. Our results suggest an early entry of the virus through the olfactory mucosa and a rapid interneuronal spread of the virus leading to acute encephalitis and neuronal damage in this mouse model.

Severe acute respiratory syndrome coronavirus 2 (SARS-CoV-2) was identified in late 2019.^46^ Since then, the virus has spread globally causing more than 260 million confirmed infections and 5.2 million deaths worldwide (World Health Organization; https://covid19.who.int/, accessed December 9, 2021). The virus has an overall 80% nucleotide identity with SARS-CoV, which was first described in China in 2002 and caused an epidemic affecting 8096 people with a case fatality of around 10%.^24^

Both SARS-CoV and SARS-CoV-2 are known to enter human host cells primarily by binding to the cellular receptor angiotensin-converting enzyme 2 (ACE2) and by the action of the transmembrane protease serine 2 (TMPRSS2) for spike (S) protein priming.^28^ ACE2 and TMPRSS2 have been shown to be expressed in the lung and bronchial epithelium as well as in nasal olfactory mucosa, consistent with respiratory disease as being the most frequent outcome of infection.^43^ Although the upper and lower respiratory tracts are the major sites of infection, SARS-CoV-2 affects multiple organ systems and many non-pulmonary symptoms are also recognized.^17^ Recently, it was also discovered that neuropilin-1 (NRP1) significantly potentiates viral infectivity especially into olfactory neuroepithelial cells in the nasal cavity.^7^ Interestingly, while ACE2 was expressed at low levels in both human lung tissue and olfactory epithelium, the expression of NRP1 was abundant in these tissues supports its role in SARS-CoV-2 entry into olfactory neuroepithelial cells. This could explain the loss of olfactory function in some Coronavirus Disease 2019 (COVID-19) patients.^18^

Previous studies have shown the ability of SARS-CoV to cause neuronal death in humanized ACE2 (hACE2)-transgenic mice by invading the brain via the olfactory epithelium.^27,31^ This might be explained by the presence of hACE2 in the brain of this animal model.^27^ As reported in humans, ACE2 is an important component of the renin-angiotensin system in the brain and is expressed by endothelial, neuronal, and glial cells of the central nervous system (CNS).^16^ Also, TMPRSS2 is co-expressed in some human brain cell types including astrocytes, oligodendrocytes, and neurons in some brain regions.^14^ Thus, it is possible that SARS-CoV-2 could enter the CNS and cause neurological damage.^23,28^ Neurological manifestations have been reported in 36.4% of patients that succumbed to COVID-19.^25^ Indeed, the expression of ACE2 in the brain is consistent with the reported central and peripheral neurological symptoms in patients who died of COVID-19 and even a possible dysfunction of brainstem cardiopulmonary regulatory centers that has been related to the sudden cessation of breathing in COVID-19 patients.^18,25^ However, the neuropathological features of COVID-19, including glial response, inflammatory changes, and the presence and distribution of SARS-CoV-2 in the brains of patients who died from COVID-19, are not well characterized yet.

Potential routes of SARS-CoV-2 neuroinvasion have been widely investigated. In general, there are 2 anatomical routes for a virus to enter the CNS, a neural pathway and through body fluids (such as blood, lymph, and cerebrospinal fluid).^23^ Like SARS-CoV,^31^ the main pathway for SARS-CoV-2 to enter the brain is through retrograde axonal transport from the olfactory nerve.^6,12,13,28^ However, the detection of SARS-CoV-2 RNA in CNS regions that have no direct connection to the olfactory mucosa, such as the cerebellum, suggests that there may be other mechanisms or routes of viral entry into the CNS, possibly in addition to or in combination with axonal transport. For instance, migration of SARS-CoV-2-carrying leukocytes across the blood-brain barrier or viral entry along CNS endothelia cannot be excluded.^23,28^ Moreover, myeloid cells are able to *trans*-infect SARS-CoV-2 to cellular targets expressing hACE2 and TMPRSS2.^35^

Although different neurological presentations related to COVID-19 have been described, there is no consensus on the consequences of CNS infection.^40^ These CNS complications range from nonspecific symptoms to necrotizing encephalopathies, encephalitis, myelitis, encephalomyelitis, vasculitis, and stroke.^3^ While several different mechanisms involved in COVID-19-associated CNS dysfunction have been postulated, the 2 main competing hypotheses are based on (1) neurotropism and direct invasion of SARS-CoV-2 into the CNS and (2) indirect mechanisms mediated by the cytokine storm induced by systemic SARS-CoV-2 infection. The cytokine storm would be followed by extensive endothelial damage, a state of hypercoagulation and thromboembolic events resulting in ischemic stroke.^26,41^

Since the original SARS-CoV-2 variant that caused the current pandemics does not efficiently bind to the wild-type mouse ACE2 receptor, transgenic mice expressing the human ACE2 (hACE2) under the keratin 18 (K18) promoter have been established as an animal model of COVID-19.^20,33^ This model was initially developed to study the SARS-CoV infection,^27,31^ for which viral invasion and replication in the brain is currently well described.^41^

The aim of the present study was to assess the kinetics of SARS-CoV-2-induced histological lesions in the brain of hACE2 mice. Here, we correlated the brain lesions with virus immunolabeling and analyzed the neuroinflammatory response induced by the virus.

## Material and Methods

### Ethics Statement

Animal experiments were approved in advance by the Institutional Animal Welfare Committee of the Institut de Recerca i Tecnologia Agroalimentàries (CEEA-IRTA, registration number CEEA 188/2020) and by the Ethical Commission of Animal Experimentation of the Government of Catalonia (registration number FUE-2020-01589810) and conducted by certified staff. Experiments with SARS-CoV-2 were performed at the Biosafety Level-3 (BSL-3) facilities of the Biocontainment Unit of IRTA-CReSA (Barcelona, Spain).

### Virus Isolates

The SARS-CoV-2 isolate used in these studies was hCoV-19/Spain/CT-2020030095/2020 (GISAID ID EPI_ISL_510689), designated as Cat01, which was isolated from a nasopharyngeal swab of a hospitalized human patient from Catalonia, Spain, in March 2020.

Compared with the Wuhan/Hu-1/2019 strain, the Cat01 isolate has the following point mutations: D614G (Spike), R682L (Spike), C16X (NSP13), and another 12 in NSP3 (M1376X, P1377X, T1378X, T1379X, I1380X, A1381X, K1382X, N1383X, T1384X, V1385X, K1386X, S1387X).^4^ The D614G mutation predicts the replacement of the aspartate residue at position 614 of the spike protein (or S-protein) by a glycine residue.^19^ The mutation has enhanced the transmissibility of the virus, with infections resulting in higher viral loads.^36^ Production of virus stocks (Cat01 passage number 3), isolation, titration, and live virus neutralization assay were performed in Vero E6 cells (ATCC CRL-1586). Virus titers were determined using a standard tissue culture median infectious dose (TCID_50_) assay and expressed as TCID_50_/mL.

### Study Design

A total of 52 transgenic B6.Cg-Tg(K18-ACE2)2Prlmn/J (K18-hACE2) mice (24 males and 28 females) were allocated into the BSL-3 facilities at (IRTA-CReSA). One week after their arrival, animals were distributed into 3 experimental groups. (1) High-dose group animals (*n* = 24; 12 males, 12 females) were challenged with 10^4^ TCID_50_/mouse SARS-CoV-2 Cat01 isolate via intranasal route (50 µL/individual, 25 µL in each nostril) under isoflurane anesthesia. (2) Low-dose group mice (*n* = 14; 9 males, 5 females) were inoculated with 10^3^ TCID_50_/animal SARS-CoV2 following the same procedure as in high-dose group. (3) The remaining animals (control group; *n* = 14; 3 males, 11 females) were mock challenged with phosphate-buffered saline solution. Animals were monitored and weighed daily. At 2, 4, 6, and 7 days postinfection (dpi), mice were sacrificed or found dead, as described in Table 1. Endpoint criteria were defined by a severe clinical score (over 15 points on a 34-point scale), including loss of >20% bodyweight, kyphosis, rough coat, depression, and respiratory and/or digestive signs, and also included qualitative criteria (presence of seizures, coma, or severe dehydration). Euthanasia was performed by intraperitoneal pentobarbital overdose under general anesthesia (5% isoflurane). Necropsies were performed, and different samples were collected for pathological evaluation at the different time points. The right half of the brain and right ethmoid turbinate were fixed by immersion in neutral buffered 10% formalin for histopathological analyses. For virological studies, the other half brain and ethmoid turbinates were transferred into cryotubes containing 500 µL Dulbecco’s modified Eagle medium (DMEM; Lonza) supplemented with 100 U/mL penicillin, 100 μg/mL streptomycin, and 2 mM glutamine (ThermoFisher Scientific) and 5-mm diameter inox beads.

**Table 1.** Number of animals found dead or euthanized following the study protocol after SARS-CoV-2 inoculation.

Dose of inoculum per mouse	Days post-SARS-CoV-2 inoculation			
	2	4	6	7
10^4^ TCID_50_ (*n* = 24)	6	6	5 (all found dead)	7
10^3^ TCID_50_ (*n* = 14)	—	7	1 (found dead)	6 (1 animal found dead)
Mock inoculated (*n* = 14)	3	7	—	4

Abbreviation: TCID_50_, tissue culture median infectious dose.

### Viral Detection and Infection Assays

For viral RNA detection and infectious virus titration assays, the left part of the collected brain was homogenized at 30 Hz for 2 minutes using a TissueLyser II (QIAGEN GmbH). Viral RNA was extracted using the IndiMag pathogen kit (Indical Bioscience) on a Biosprint 96 workstation (Qiagen) according to the manufacturer’s instructions. Real-time reverse transcription quantitative PCR (RT-qPCR) was used to detect viral genomic and subgenomic RNA (gRNA and sgRNA, respectively, where subgenomic RNAs are generated after cell entry and generation of transcripts, and provide a measure of replicative virus) to differentiate inactivated virus from productive infection according to the previously published protocol^11,45^ with minor modifications to adapt it to the AgPath-ID One-Step RT-PCR Kit (Life Technologies). Details of the technique, including primers and probes, are found elsewhere.^4^

Brain samples positive by RT-qPCR were titrated in Vero E6 cells (ATCC CRL-1586), and incubated at 37 °C and 5% CO_2_ for 6 days, as previously described.^4^ Briefly, samples were 10-fold diluted, transferred in a 96-well plate with Vero E6 cells monolayer, and incubated at 37 °C, 5% CO_2_. Duplicates were made for each sample. Plates were evaluated for the presence of cytopathic effect at 5 to 6 dpi. The amount of infectious virus was calculated by determining the TCID_50_ using the Reed-Muench method.

### Pathology and Immunohistochemistry

Routine formalin-fixed paraffin-embedded tissue samples from the nasal cavity (olfactory mucosa) and brain were used for this study. Nasal cavity samples were decalcified after fixation for 4 hours at room temperature in a hydrochloric acid (5% to 10%) and formic acid (2% to 5%) rapid decalcifying solution. These samples were sectioned to analyze the nasal mucosa and the brain. The latter was cut into transverse sections to examine the olfactory bulb, telencephalon, diencephalon, mesencephalon, pons, cerebellum, and medulla oblongata. The samples were processed and stained with hematoxylin and eosin using standard laboratory procedures.

An immunohistochemistry (IHC) technique to detect SARS-CoV-2 NP antigen^38^ using the rabbit monoclonal antibody (40143-R019, Sino Biological) at dilution 1:15 000 was applied on nasal turbinates, olfactory bulb, and brain sections of all animals of the 3 experimental groups. As a positive control, formalin-fixed lung sections from a SARS-CoV-2-infected macaque, kindly supplied by Prof Bart L. Haagmans, Erasmus Medical Center, Rotterdam, the Netherlands, were used to set up the technique in mice. See Supplemental File 1 for further procedural details.

To characterize histological findings in the CNS, IHC of brain samples was also done only in 12 animals at 4 and 7 dpi (8 from low-dose group, 4 each day; 4 from control group, 2 each day). Antibodies against the following antigens were used: glial fibrillary acidic protein (GFAP, rabbit polyclonal, Dako Z0334; dilution 1:2000) to label astrocytes; ionized calcium binding adaptor molecule 1 (IBA1, goat polyclonal, ABCAM ab5076; dilution 1:300) to label microglia; CD3 (rabbit monoclonal IgG anti mouse CD3-epsilon clone D4V8L, Cell Signalling; dilution 1:500) to label T cells and CD20 (goat polyclonal IgG anti mouse CD20, Santa Cruz Biotechnology, SC-7735; dilution 1:250) to label B cells. See Supplemental File 1 for further procedural details.

The EnVision+ System linked to horseradish peroxidase (HRP, Agilent-Dako) and 3,3′-diaminobenzidine (DAB) was used as a chromogen substrate for all antibodies, except for CD20 and IBA1. For CD20, ImmPRESS-AP-alkaline phosphatase linked-horse anti-goat IgG Polymer (Maraval Life Sciences, Vector Laboratories) and a red chromogen were used. For IBA1, a polyclonal rabbit anti-goat biotinylated secondary antibody (DAKO, 1:200) with the ABC Peroxidase Standard Staining Kit (Thermo Scientific) and DAB as a chromogen were used. Finally, slides were counterstained with hematoxylin.

The amount of viral antigen in tissues was semiquantitatively scored in the different brain areas as (0) lack of, (1) low, (2) moderate, or (3) high amount of immunolabeled cells.^4^ The degree of astrogliosis and microgliosis was also semiquantified as none (0), slight (1), moderate (2), marked (3), or severe (4) for GFAP as an astrocyte marker and IBA1 as a marker for microglial cells. The following brain areas were evaluated, when present, and a score was given to the whole area: olfactory mucosa, olfactory bulb, piriform cortex, hippocampus, striatum, frontal cortex, parietal cortex, occipital cortex, temporal cortex, thalamus, hypothalamus, mesencephalon, pons, medulla oblongata, and cerebellar cortex. Supplemental File 2 (S2) shows the scores for each mouse studied.

### Statistical Analyses

Statistical analyses were completed using GraphPad Prism 8. Mean weight data from different groups per study day were analyzed by multiple unpaired *t* test. Normality of each data set for gRNA, sgRNA, and infection viral loads was calculated using the Shapiro-Wilk test. Comparison of viral loads between animal groups was performed with multiple unpaired *t* test. In all analyses, a *P* value <.05 was considered statistically significant.

## Results

### hACE2 Mice Develop Neurological Signs Upon Infection With SARS-CoV-2

**Figure 1.**
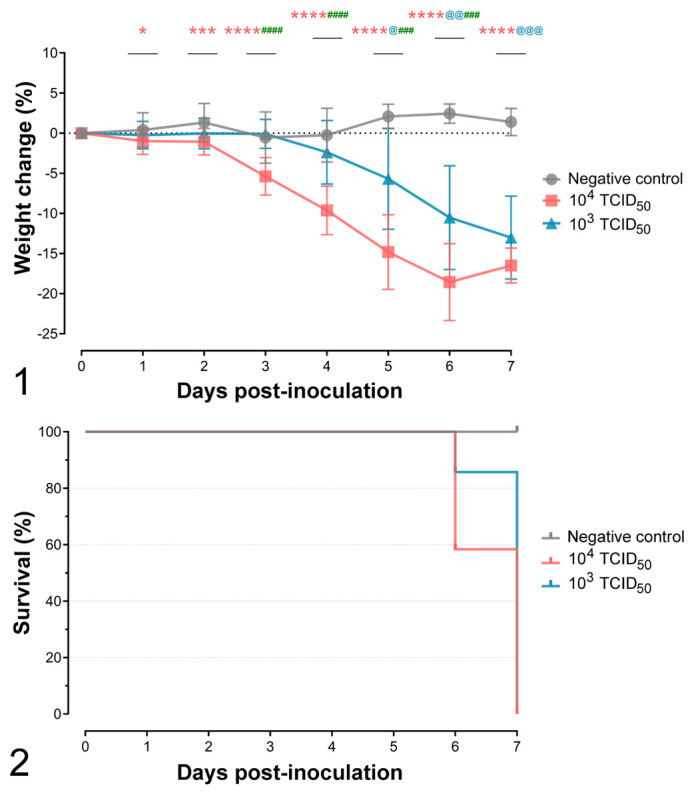
Progression of weight loss after inoculation of hACE2-transgenic mice with either 10^3^ or 10^4^ SARS-CoV-2 TCID_50_ per animal. Significant differences between groups are shown as asterisks above each time point. Red asterisks show differences between the 10^4^ TCID_50_-inoculated group and noninfected animals. Blue asterisks show differences between 10^3^ TCID_50_-inoculated group and noninfected animals. Green asterisks show differences between 10^4^ and 10^3^ TCID_50_-inoculated groups. ns, *P* > .05; *, *P* ≤ .05; **, *P* ≤ .01; ***, *P* ≤ .001; ****, *P* ≤ .0001. The bars show the mean ± standard deviation. **Figure 2.** Survival curves of hACE2-transgenic mice inoculated with either 10^3^ or 10^4^ TCID_50_ per animal of SARS-CoV-2 and the negative controls.

No overt clinical signs were observed in mice up to 5 dpi, except for weight loss starting from 3 dpi onwards (Fig. 1). Five animals that received the higher SARS-CoV-2 dose had to be euthanized at 6 dpi due to final endpoint criteria. From 5 to 7 dpi, all animals displayed apathy, depression, hunched posture, and, when touched, neurological signs consisting of trembling and seizures. A survival curve of studied animal groups is displayed in Figure 2.

### SARS-CoV-2 Loads in Brain of hACE2 Mice

**Figures 3–5.**
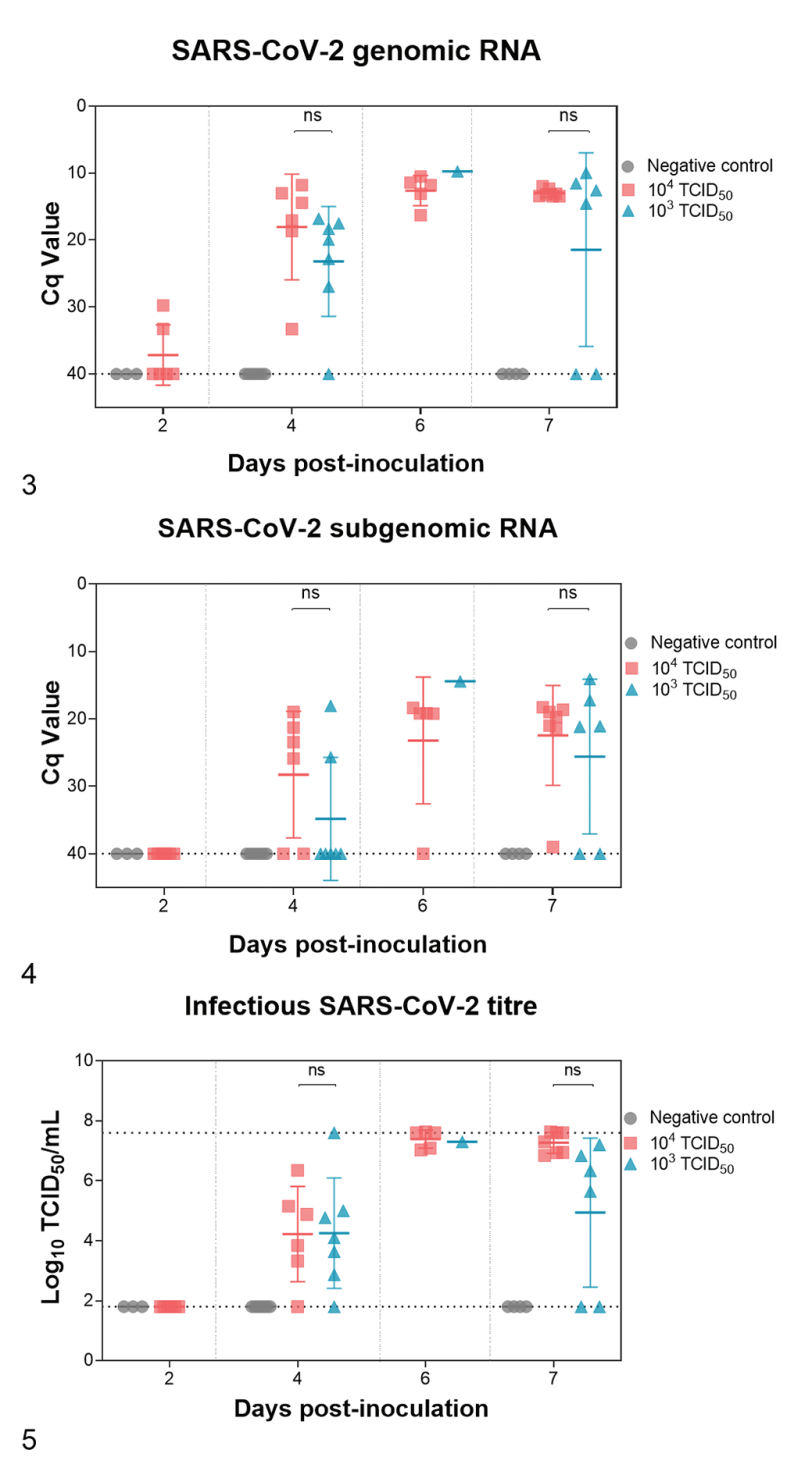
Virological studies. No significant differences in viral loads in brain were observed between the 2 dose groups at 4 and 7 days postinoculation (dpi). In the high-dose group, viral genomic DNA was found in the brain as early as 2 dpi. **Figure 3.** Viral load in the brain of SARS-CoV-2-inoculated hACE2-transgenic mice measured by means of viral genomic RNA. **Figure 4.** Viral load in the brain of SARS-CoV-2-inoculated hACE2-transgenic mice measured by means of viral subgenomic RNA. **Figure 5.** Viral load in the brain of SARS-CoV-2-inoculated hACE2-transgenic mice measured by means of infectious virus (in cell culture). Each dot represents a single animal. ns, nonsignificant differences. The bars show the mean ± standard deviation.

The presence of SARS-CoV-2 was monitored in brain samples collected during necropsies. gRNA was barely detected at 2 dpi in the high-dose group (Fig. 3), while no sgRNA (indicative of viral active replication) was found at this early stage (Fig. 4). Although not significant, slightly higher viral RNA loads were noticed in the 10^4^ TCID_50_- compared to the 10^3^ TCID_50_-inoculated group at 4 dpi. From 4 dpi until the end of the study, high levels of gRNA and sgRNA were quantified from both high- and low-dose group. No SARS-CoV-2 RNA was detected in animals from the negative control group.

In line with RT-qPCR results, no infectious virus was found at 2 dpi (based on the isolation on Vero E6 cells). At later time points, moderate amounts of SARS-CoV-2 were detected independently of the inoculated dose (Fig. 5). Higher amounts of virus were detected in both high- and low-dose groups at 6 and 7 dpi, when the mice had to be euthanized prior to the planned endpoint. Although no statistical differences between groups were observed, the infectious SARS-CoV-2 titers were especially high in the 10^4^ TCID_50_-inoculated animals.

### SARS-CoV-2 Induces Nonsuppurative Meningoencephalitis, Thrombosis, and Vascular Damage

No lesions were observed in the brain of any animal on 2 dpi infected with 10^4^ TCID_50_. At 4 dpi, lesions were observed in a few SARS-CoV-2-inoculated animals (2 animals that received 10^3^ TCID_50_ and 3 animals receiving 10^4^ TCID_50_) and later, at 7 dpi, in approximately 50% of the animals inoculated with 10^3^ TCID_50_ dose and all animals inoculated with the higher dose (Table 2). The severity and extent of the lesions was higher in the high-dose group.

**Table 2.** Kinetics of lesion development in the brain of mice following inoculation with SARS-CoV-2.

Dose of inoculum per mouse	Histopathological finding	Days after SARS-CoV-2 inoculation			
		2^a^	4^a^	6	7^b^
10^4^ TCID_50_ (*n* = 22)	Meningoencephalitis	0/5	2/5	5/5	7/7
	Vasculitis	0/5	1/5	3/5	7/7
	Thrombosis	0/5	0/5	3/5	7/7
	Hemorrhages	0/5	2/5	3/5	7/7
	Neuronal vacuolar degeneration	0/5	0/5	5/5	7/7
	White matter tract myelin sheath vacuolation	0/5	0/5	5/5	7/7
10^3^ TCID_50_ (*n* = 14)	Meningoencephalitis		2/7		4/7
	Vasculitis		2/7		3/7
	Thrombosis		1/7		3/7
	Hemorrhages		1/7		4/7
	Neuronal vacuolar degeneration		0/7		3/7
	White matter tract myelin sheath vacuolation		1/7		5/7

Abbreviation: TCID_50_, tissue culture median infectious dose.

^a^One animal of each time point of the 10^4^ group was not included in the histopathological study because the whole brain was allocated to virological experiments.

^b^ One animal of the 10^3^ dose group was found dead at 6 dpi and has been included in the 7 dpi group.

**Figures 6–11.**
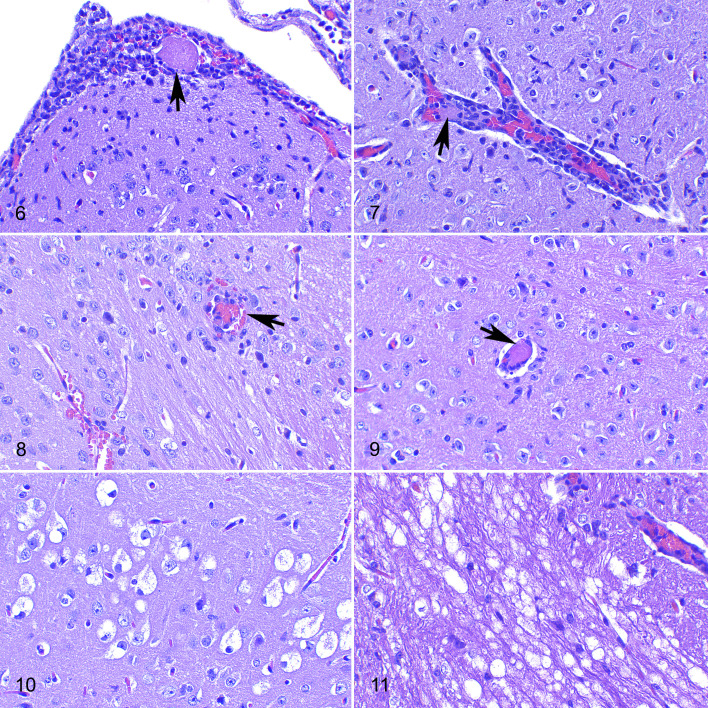
SARS-CoV-2 infection, brain, hACE2-transgenic mice, at 7 days postinoculation, 10^4^ TCID_50_ dose, hematoxylin and eosin. **Figure 6.** Occipital cortex. Mononuclear cell infiltrate in the leptomeninges with a thrombus in a blood vessel (arrow). **Figure 7**. Mesencephalon. A perivascular cuff of mononuclear cells, and a thrombus within a blood vessel. **Figure 8.** Occipital cortex, gray to white matter transition. There is vasculitis (arrow) and small hemorrhages (bottom left). **Figure 9**. Thalamus. Intravascular thrombus (arrow) in a small blood capillary. **Figure 10.** Hippocampal formation. Vacuolar degeneration of neurons in the dorsal end of the subiculum. **Figure 11.** Medulla oblongata. Myelin sheath vacuolation, limited to the root of the facial nerve.

Lesions consisted of nonsuppurative meningitis, encephalitis, or a combination of both (meningoencephalitis). These lesions were characterized by a leptomeningeal infiltrate of mononuclear cells (Fig. 6). This infiltrate was also observed surrounding small capillaries (perivascular cuffing) in both gray and white matter, with an increased cellularity of the brain parenchyma interpreted as gliosis (Fig. 7) and mild to moderate neuropil spongiosis. At 4 dpi, these lesions were confined to the piriform cortex, thalamus, and mesencephalon, whereas at 6 to 7 dpi, when observed, the lesions were widespread in all brain regions studied. Lesions in the cerebellar cortex were milder than in other areas, consisting mainly of mild meningitis and spongiosis.

In addition to these inflammatory infiltrates, vascular alterations were observed at 4, 6, and 7 dpi in animals infected with the high viral dose and also at 7 dpi in low-dose infected animals. Vascular damage was mainly seen in small blood vessels throughout the gray and white matter and was characterized by a mixture of neutrophils, lymphocytes, and few plasma cells expanding the vascular wall (vasculitis) and, occasionally, extending into the perivascular (Virchow-Robin) spaces (Fig. 8). Numerous capillaries were occluded by fibrin thrombi (thrombosis; Figs. 6, 7, and 9) and adjacent tissue sometimes had microhemorrhages (Fig. 8). However, ischemic necrosis was not observed. Vascular lesions were more severe and frequent in the high-dose animals sacrificed at 7 dpi compared to the mice receiving the lower dose on the same dpi.

Mock infected controls did not show any of the abovementioned changes at any time.

### Early Lesions of SARS-Cov-2-Induced Neuronal Damage

Clusters of swollen neurons with foamy or vacuolated cytoplasm (cytoplasmic ballooning), occasionally with pyknotic and eccentric nuclei, were observed without an associated neuroinflammatory reaction (Fig. 10). This finding was observed only at 6 and 7 dpi and was present in all high-dose infected animals and also, albeit less severely, in a few low-dose infected animals. Mock infected controls did not show this change at any time.

The above-mentioned degenerating neurons were observed in the same locations in the brain of different mice, mainly in the hippocampus and the frontal, parietal, occipital, and temporal lobes, mostly involving layers IV and V of the neocortex. In the hippocampus, these neurons were consistently located in the subiculum. In the occipital lobe, they were located within the presubiculum area. In the frontal cortex, they were mainly located in one or more areas from the primary somatosensory cortex and/or the primary or secondary motor cortices. In the parietal cortex they were located within retrosplenial agranular and granular cortex. Finally, in the temporal cortex, degenerating neurons were located within the primary and secondary auditory and visual cortex areas. Occasionally, similar changes were observed in isolated neurons in the mesencephalon tegmentum, pons, and medulla oblongata but not in the cerebellum.

Intense bilateral and symmetrical myelin sheath vacuolation was also seen in the white matter tracts of the pons and the brainstem of animals at 7 dpi, both at low and high doses. In mice inoculated with the higher dose of virus, these findings were more frequent and were mostly located in both the sensory root of the trigeminal nerve in the pons area and spinal trigeminal tract, in the vestibular root of the vestibulocochlear nerve, and in the facial nerve and its root in the medulla oblongata (Fig. 11).

### Early Detection of Virus Antigen in Olfactory Mucosa

**Figure 12.**
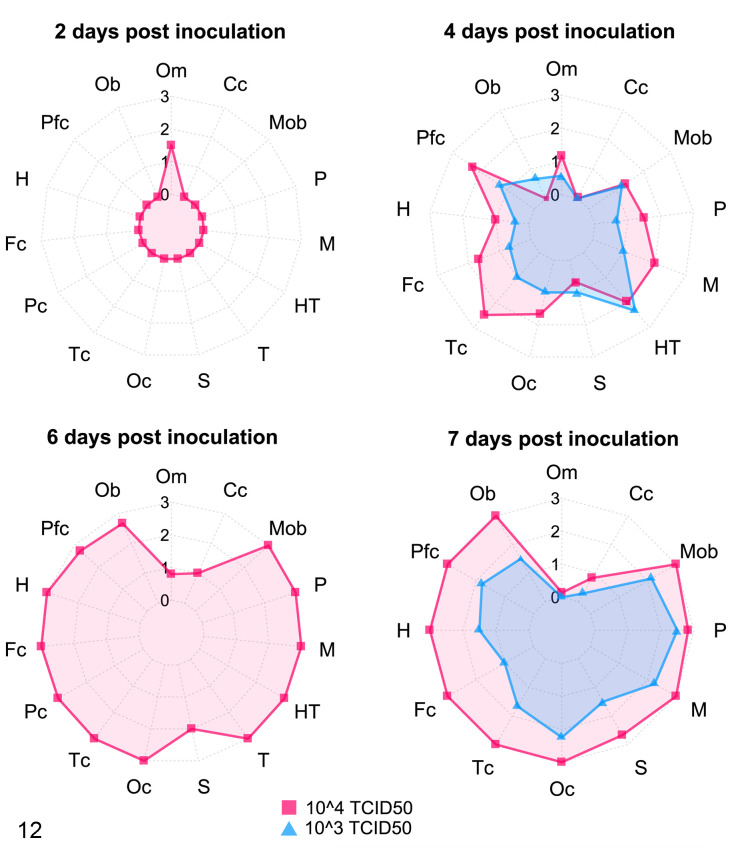
SARS-CoV2 immunolabeling in the nervous system of hACE2-transgenic mice. Mice were inoculated with 10^3^ (blue; euthanized at 4 and 7 dpi) or 10^4^ (pink; euthanized at 2, 4, 6, and 7 dpi) TCID_50_ of SARS-CoV-2. Different areas of the brain and nasal cavity were semiquantitatively scored from 0 (no immunolabeling) to 3 (maximum immunolabeling) and the mean scores in each group are shown. Levels from 0 to 3 are shown by concentric circles, with the outside circle being 3. At 2 dpi, virus was only found in the olfactory mucosa, but at 4 dpi the virus had reached most brain areas (for both doses of virus). Virus throughout the brain was maximal at 6 and 7 dpi, with the exception of the cerebellum which is almost spared. At 7 dpi, the virus is no longer observed in the olfactory mucosa. See individual scores in Supplemental File S2. Each axis of the star-graph represents a studied region (Om, olfactory mucosa; Cc, cerebellar cortex; Mob, medulla oblongata; P, pons; M, mesencephalon; HT, hypothalamus; T, thalamus; S, striatum; Oc, occipital cortex; Tc, temporal cortex; Pc, parietal cortex; Fc, frontal cortex; H, hippocampus; Pfc, piriform cortex; Ob, olfactory bulb).

**Figures 13–18.**
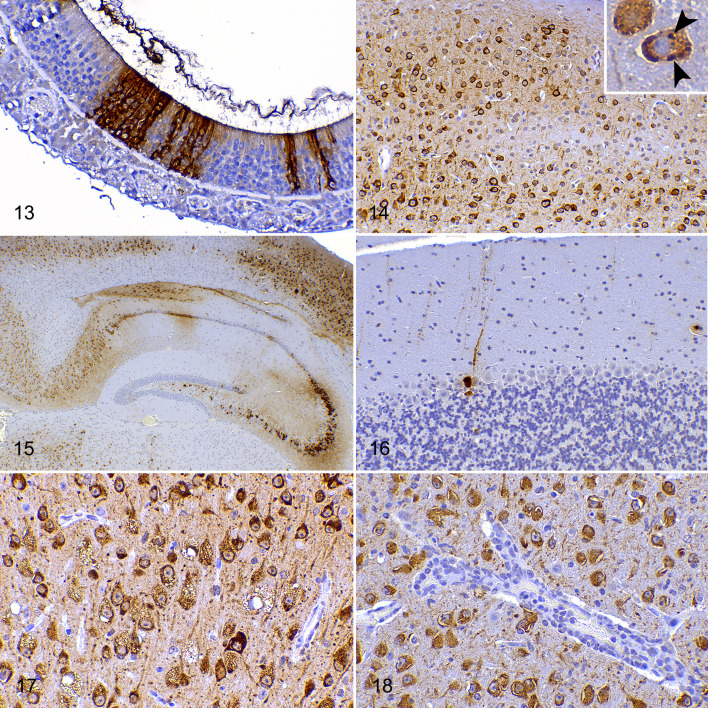
SARS-CoV-2 infection, olfactory mucosa (Fig. 13), and brain (Figs. 14–18), hACE2-transgenic mice, immunohistochemistry for SARS-CoV-2. **Figure 13.** Small clusters of immunolabeled cells in the olfactory epithelium (2 dpi, 10^4^ TCID_50_ dose). **Figure 14.** Widespread intracytoplasmic neuronal immunolabeling in all layers of the occipital cortex (7 dpi, 10^4^ TCID_50_ dose). Inset: immunolabeled cytoplasmic inclusion bodies in a neuron (arrowheads). **Figure 15.** The early distribution of SARS-CoV-2 antigen immunolabeling with patchy involvement of thalamus, CA1, CA2, CA3, and subiculum of the hippocampus (the lateral portion of CA1 and the dentate gyrus are mostly spared) and the parietal cortex (4 dpi, 10^4^ TCID_50_ dose). **Figure 16.** The cerebellar cortex is mostly spared, and only the perikaryon and dendrites of a few piriform (Purkinje) cells are immunolabeled (7 dpi, 10^4^ TCID_50_ dose). **Figure 17.** Parietal cortex. All degenerating neurons with cytoplasmic vacuoles are immunolabeled (7 dpi, 10^4^ TCID_50_ dose). **Figure 18**. Mesencephalon. There is immunolabeling of neurons but not of the endothelium in a vessel that has a cuff of mononuclear cells (the same blood vessel depicted in Fig. 7; 7 dpi, 10^4^ TCID_50_ dose).

At 2 dpi, clusters of cells of the olfactory mucosa, including those with features compatible with neuroepithelial cells, were immunolabeled for SARS-CoV-2 in the high-dose infected group. The infected cells were characterized by diffuse cytoplasmic labeling. At 2 dpi, no SARS-CoV-2 immunolabeling was observed in the olfactory bulb or the other CNS areas (Figs. 12, 13). Viral antigen was also observed in the olfactory mucosa at 4 and 6 dpi, although in fewer cells, but it was no longer observed in the olfactory mucosa of animals euthanized at 7 dpi.

### Rapid Interneuronal Spread of the Virus Antigen Throughout the Brain

Multifocal clusters of neurons immunolabeled for SARS-CoV-2 NP antigen were observed at 4 dpi in high- and low-dose groups throughout the brain. At 6 and 7 dpi, the virus immunolabeling had extended to almost all evaluated brain areas, regardless of the infective dose, with its maximal distribution throughout the brain at 7 dpi (Fig. 12) and with higher immunohistochemical scores given to the high-dose group. The immunolabeling pattern consisted of a diffuse to finely granular intracytoplasmic staining of the perikaryon and the neurites (Figs. 14, 15) of cells morphologically identified as neurons. Occasionally, more intensely stained rounded inclusion bodies were observed (Fig. 14). Only the cerebellum was spared to a certain extent since, even in the high-dose group at 7 dpi, only a few neurons were immunolabeled; these neurons were morphologically identified as cerebellar piriform (Purkinje) cells (Fig. 16).

A significant number of morphologically normal neurons were labeled for viral protein, while all neurons showing cytoplasmic ballooning contained SARS-CoV-2 antigen (Fig. 17). Occasional amoeboid-shaped histiocytes with labeled cytoplasm could be observed in the subarachnoidal space or perivascular cuffs. However, no other cell types were immunolabeled including astrocytes, oligodendroglial cells, ependymal cells, choroid plexus epithelium, or leptomeningeal cells. Endothelia and inflammatory cells in perivascular cuffs (Fig. 18) were not immunolabeled, including those present within lesions of vasculitis or thrombosis (except for the odd macrophage mentioned earlier).

### SARS-CoV-2 Induces Strong Astroglial and Microglial Responses and More Limited T-Cell and B-Cell Infiltrates

**Figures 19–20. fig6-03009858211066841:**
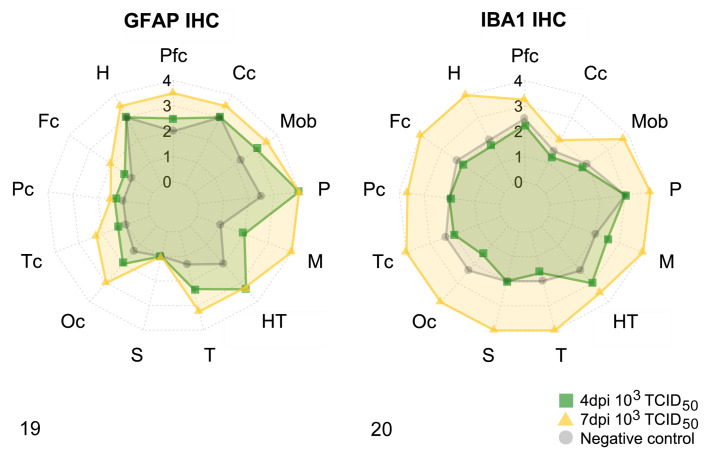
Quantification of the neuroinflammatory response in brains of hACE2-transgenic SARS-CoV-2-infected mice (10^3^ TCID_50_ dose) at 4 dpi and 7 dpi, compared to mock-inoculated negative controls. Different areas of the brain were semiquantitatively scored from 0 (no immunolabeling) to 4 (maximum immunolabeling) and the mean scores in each group are shown. Levels from 1 to 4 are shown by concentric circles, with the outside circle being 4. Each axis of the star-graph represents a brain region; the value given is the average score for all the animals in that group in that particular area (Pfc, piriform cortex; Cc, cerebellar cortex; Mob, medulla oblongata; P, pons; M, mesencephalon; HT, hypothalamus; T, thalamus; S, striatum; Oc, occipital cortex; Tc, temporal cortex; Fc, frontal cortex; Pc, parietal cortex; H, hippocampus). **Figure 19**. Scoring of GFAP immunolabeling. Astrogliosis (hypertrophy and hyperplasia of astrocytes) is most evident in the brainstem (thalamus, hypothalamus, mesencephalon, pons, and medulla oblongata), piriform cortex, and occipital cortex, and is evident at 4 dpi (green squares) but more intense at 7 dpi (yellow triangles). The hippocampus, parietal cortex, striatum, and cerebellar cortex show small or no differences compared to uninoculated negative controls (gray circles). **Figure 20**. Scoring of the IBA1 immunolabeling. Widespread microgliosis is most severe at 7 dpi (yellow triangles), but was similar at 4 dpi (green squares) as in the negative controls (gray circles). Microglial spares only the cerebellar cortex. See individual scores in Supplemental Table S2.

Immunophenotyping of the neuroinflammatory response to the viral infection was carried out using IHC against GFAP (astroglial cells) and IBA1 (microglial cells; Figs. 19 [Fig fig6-03009858211066841] [Fig fig7-03009858211066841] [Fig fig7-03009858211066841]–23), CD3 (T-lymphocytes), and CD20 (B-lymphocytes; Fig. 24a and b).

**Figures 21–23. fig7-03009858211066841:**
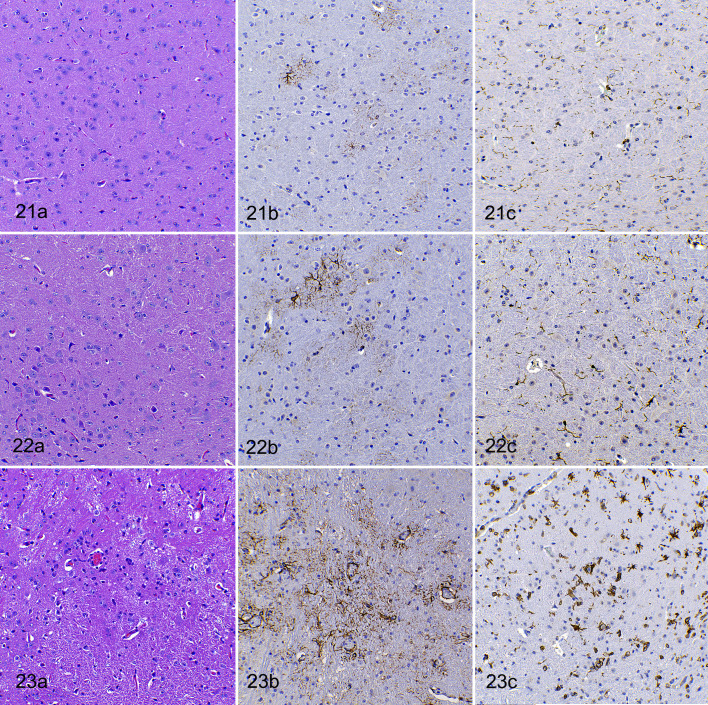
SARS-CoV-2 infection (10^3^ TCID_50_ dose), brain (mesencephalon), hACE2-transgenic mice. (a) Hematoxylin and eosin (HE), (b) immunohistochemistry (IHC) for GFAP, and (c) IHC for IBA1. **Figure 21**. Negative control mouse (mock-inoculated). **Figure 22.** SARS-CoV-2-infected at 4 dpi. (a) The increase in cellularity is not striking in HE-stained sections. (b) Moderate increase in number and hypertrophy of astroglial cells. (c) Moderate increase in number and activation of microglial cells. **Figure 23.** SARS-CoV-2-infected at 7 dpi. (a) Markedly increased cellularity. (b) Marked increase in number and size of astrocytes near vessels and in neuropil. (c) Marked increase in number and size of microglial cells.

**Figure 24.**
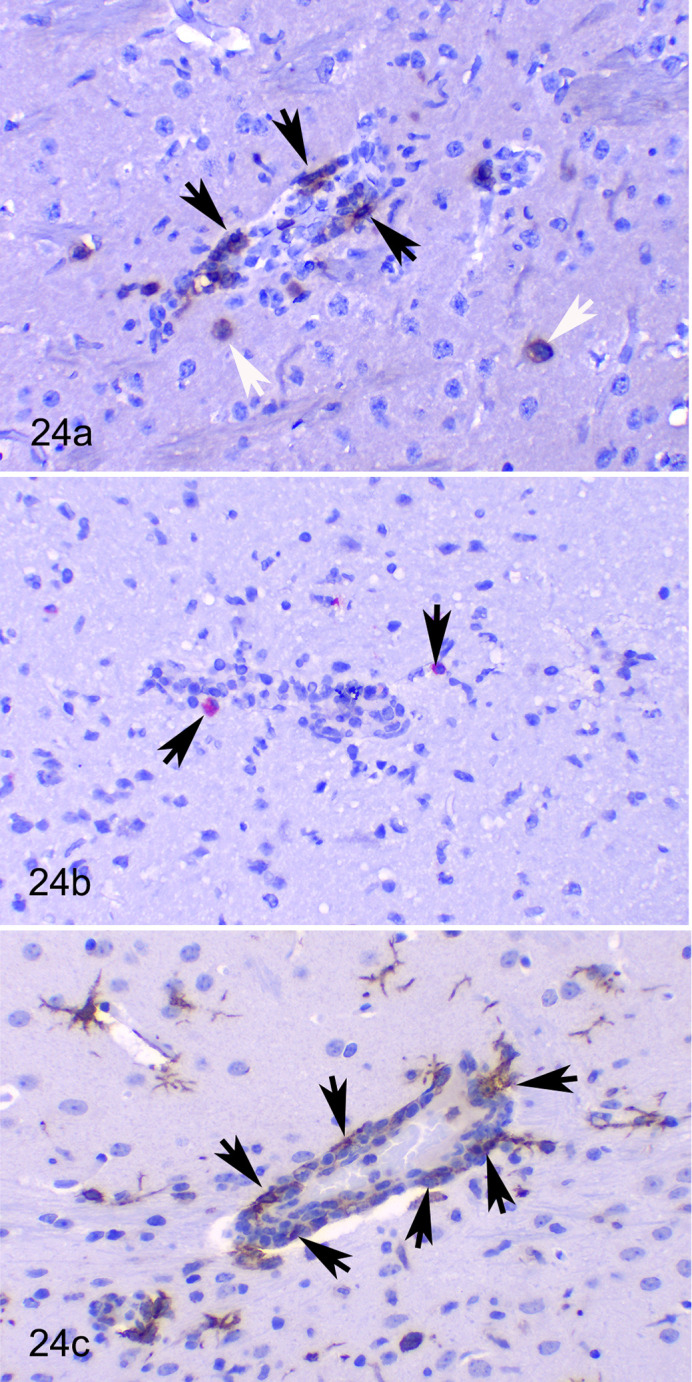
SARS-CoV-2 infection (10^4^ TCID_50_ dose, 7 dpi), brain (mesencephalon), hACE2-transgenic mice. Immunohistochemistry. (a) Several CD3-positive cells (T cells) are in most perivascular cuffs (black arrows) and occasional T cells infiltrate the neuropil (white arrows). (b) Comparatively, there are low numbers of CD20-positive cells near vessels and in neuropil (B cells, arrows). (c) Numerous IBA1-positive cells (macrophages) are observed in perivascular cuffs (arrows). IBA1-positive activated microglial cells are present in the neuropil.

GFAP IHC was scored only on 4 and 7 dpi for 10^3^ TCID_50_ challenged animals. This revealed a prominent astrogliosis at both time points, but greater intensity at 7 dpi (Figs. 19,21b [Fig fig7-03009858211066841]–23b). Astrocyte hypertrophy and hyperplasia were most noticeable in the piriform and occipital cortices and in the brainstem (thalamus, hypothalamus, mesencephalon, pons, and medulla oblongata). In 2 animals at 7 dpi, a conspicuous hyper-ramified astrocyte phenotype was observed (Fig. 23b).

IBA1 IHC revealed increased numbers of hypertrophied (activated phenotype) microglial cells only in animals at 7 dpi, regardless of the inoculated dose of virus. Microgliosis was scored only at 4 and 7 dpi in mice that received the lower dose (Figs. 20, 21c [Fig fig7-03009858211066841]–23c). This was barely noticeable at 4 dpi but was established in all animals at 7 dpi throughout all the brain areas studied, except for the cerebellum.

Perivascular cuffs and meningeal inflammatory infiltrates were composed mostly of IBA1 positive macrophages (Fig. 24c) as well as CD3-positive T lymphocytes (Fig. 24a) and occasional CD20 positive B-lymphocytes (Fig. 24b). A few T lymphocytes were also observed infiltrating the brain parenchyma.

## Discussion

As previously described for SARS-CoV^31^ and SARS-CoV-2,^1,15,20,21,33,39^ the hACE2-transgenic mice were fully susceptible to infection and disease progression with the SARS-CoV-2 isolate used in the present study. As expected, the animals developed a fatal clinical course of disease in 6 to 7 days, in a dose-dependent manner, with significant lesions in lung (data not shown) and brain. The clinical, virological, and pathological course of hACE2-transgenic mice infected with SARS-CoV-2 is not able to recapitulate the severe respiratory COVID-19 signs observed in humans.^20,21,33,39^ However, this animal model represents an excellent tool for testing antiviral drugs, antibodies, and vaccines, since protection against neurological disease may represent a good readout for successful product candidates.

In any case, and in addition to the respiratory symptoms, neurological complications are also common in COVID-19 human patients.^25^ In fact, neuropathological lesions compatible with a viral encephalitis^3,5,41^ or myelitis^42^ have been reported in COVID-19 patients, and a neurogenic pathway has been suggested for respiratory failure in severe COVID-19 cases.^22^ In the absence of specific animal models of SARS-CoV-2 neuroinvasion, the characterization of the kinetics and pathogenesis of the neuropathological phenotype of the transgenic hACE mice model may offer important clues to understand some of the features observed in COVID-19 patients suffering from neurological disorders.

At the earlier time point after infection, 2 dpi, viral antigen was solely found in the olfactory mucosa. At 4 dpi, in both dose groups, the virus was already found in cells morphologically compatible with neurons throughout most of the brain regions studied, regardless of the dose used in the challenge. The olfactory pathway has been suggested as a possible route for neuroinvasion of coronaviruses and it could offer a possible explanation for the olfactory and taste abnormalities reported in some COVID-19 patients.^6,12,13,28^ Other authors have discussed the role of nervus terminalis as an alternative entry route to the olfactory nerve, since several areas other than the olfactory bulb are involved at early stages of neuroinvasion.^2^ The nervus terminalis, also known as the cranial nerve 0 or cranial nerve N, consists of unmyelinated fibers that pass through the cribriform plate, medial to olfactory nerve fibers, and end within the nasal mucosa.^44^ These reports suggest that the presence of the ACE2 receptor in the nervus terminalis neurons, but not in the ones in the olfactory mucosa, explains its role as a more plausible neuroinvasion pathway. Further studies including earlier time points and a more detailed sampling of the brain and olfactory pathways are needed to corroborate these hypotheses. Another entry route that cannot be excluded is through the cranial nerves innervating the lungs or the digestive tract,^9^ or by viral entry through CNS endothelia.^19,25^ Indeed, viral antigen could be observed in the brainstem as early as 4 dpi in both inoculated dose groups. Also, infection spread to CNS by SARS-CoV-2-carrying leukocytes crossing the blood-brain barrier cannot be excluded since myeloid cells could potentially *trans-*infect SARS-CoV-2 to cellular targets of the brain expressing hACE2 and TMPRSS2.^35^

Viral antigen was widely observed, most often within neurons. The LacZ reporter associated with the cytokeratin18 promoter (K18mlacZ, used in this transgenic mouse model for hACE2 expression) is described to be constitutively expressed in brain cells.^10^ Indeed, SARS-CoV-2-infected neurons express hACE2 in this model as demonstrated by in situ hybridization experiments^8,15^ Also, studies performed in human brain organoids have demonstrated the neurotropism of SARS-CoV-2 despite a lower expression of the ACE2 receptor and TMPRSS2 when compared to lung epithelial cells.^32,37^ Other studies have reported the absence of co-immunolabeling of viral antigen in GFAP immunolabeled astrocytes^8,15^ and in IBA1 immunolabeled microglia.^8^ Indeed, very few cells (except for the occasional macrophage-shaped glial cells) compatible with glial cells were immunolabeled, suggesting a mainly trans-synaptic spread of the virus between neurons. In the same line, neuron-to-neuron axonal transport of coronaviral particles (human coronavirus OC43) has been demonstrated in vitro in neuronal cell cultures.^13^ There was no evidence of particularly higher amounts of viral antigen around ventricles, beneath leptomeninges or around circumventricular organs, which have more permeable endothelium, that would if present have potentially indicated other neuroinvasion routes such as diffusion through cerebrospinal fluid or from the blood. Surprisingly, no SARS-CoV-2 immunolabeling was observed in choroid plexus epithelial cells, a cell type that has been described to be susceptible to viral infection in human brain organoids.^32^ This pattern further supports the possibility of neuron-to-neuron spread, either from olfactory neurons or those of the nervus terminalis.

Both viral antigen immunolabeling and lesions were minimal or absent in the cerebellum of infected mice at all analyzed time points, including the late stage of acute infection when areas anatomically connected with the cerebellar cortex such as the pons or the cerebellar nuclei displayed abundant viral antigen by IHC. A similar result was reported in the same model for SARS-CoV infection,^27^ suggesting a selective lack of neurotropism of the virus toward cerebellar cortex neurons or a potential lack (or low density) of cell receptors in the cerebellar neurons. This needs to be assessed in further studies.

The SARS-CoV-2 neuroinvasion, as evidenced by antigen immunolabeling, is followed by a generalized neuroinflammatory process initially involving an astroglial response (present already at 4 dpi), which precedes a later activation of microglia throughout the brain, except for the cerebellum. Inflammatory infiltrates of lymphocytes (meningoencephalitis) were observed in a few animals at early stages (4 dpi) but in all 10^4^ TCID_50_-inoculated animals at 6 and 7 dpi and in most of the animals in the low-dose group. Similarly, vascular lesions including vasculitis and thrombosis were observed in some infected animals, more frequently in those euthanized at later time points and inoculated with higher amounts of virus.

It is unclear whether a generalized hyperinflammatory state or cytokines released by the activated microglia and/or infiltrating lymphocytes are responsible for the observed vascular lesions. SARS-CoV-2 antigen was not observed in endothelial cells at any time point nor was it associated with the presence of thrombi and vasculitis, which if present could have suggested a direct virus-related endothelial damage. However, the brain areas with little or no viral antigen (ie, cerebellar cortical lobes and vermis) were the areas with the lowest presence of such vascular lesions, suggesting they may be related. The presence of thrombi, vasculitis, or microhemorrhages was not associated with conspicuous parenchymal necrosis, indicating that they may not be caused by or result in ischemia. The fact that both the vascular lesions and gliosis were less severe in the cerebellum, where little viral antigen was seen by IHC, suggests that the presence of the virus may be related causally with such changes.

Considering the wide distribution of viral antigen in the neurons, the severity of the observed lesions was rather limited, and mostly viral antigen-loaded neurons appeared morphologically intact. However, unlike recent reports from other groups working with the same experimental model in which no neuronal damage was observed,^39^ evidence suggestive of virus-induced neuronal damage was found in mice of the present study. This feature was characterized by ballooning of neuronal perikarya in clusters of mostly cerebrocortical SARS-CoV-2 immunolabeled neurons as well as scattered neurons in the brainstem. Vacuolation of neuronal cytoplasm is indicative of neurodegeneration, and the presence of viral antigen in all the neurons with this change and its absence in mock-inoculated control mice suggests that it is a virus-induced neurodegenerative change. A similar finding was reported by Carossino and collaborators.^8^

Spongiosis of certain cranial nerve tracts of the medulla oblongata, including the sensory root of the trigeminal nerve and the spinal trigeminal tract, was also observed in infected mice, although axons in these tracts were devoid of SARS-CoV-2 antigen. This could be related to the involvement of sensitive neurons in the trigeminal ganglion, but this structure has not been evaluated in the present study and needs further assessment. Indeed, there is evidence of the involvement of the trigeminal nerve function in COVID-19 patients.^29,34^

Notably, viral load measured by different techniques (genomic and subgenomic qPCR, and infectious virus titration) unequivocally followed the same pattern described by the IHC in terms of viral antigen detected in brain histological sections. Infectious virus in the brain was detected from 4 dpi onwards in an increasing trend, with the highest loads detected by 6 and 7 dpi, as expected.^30^ Interestingly, no significant difference in terms of viral load was observed in the brain at the latest stage regardless of the viral inoculum dose.

In summary, hACE2 mice were fully susceptible to SARS-CoV-2 infection, causing a fatal outcome characterized by neurological clinical signs and mild to moderate brain lesions. After an early infection of olfactory mucosa, the virus quickly spread, probably neuron-to-neuron, through most brain areas (except the cerebellum) causing nonsuppurative meningoencephalitis, thrombosis, microhemorrhages, and vasculitis. These lesions were mainly seen 6 to 7 dpi, when the final endpoint criteria were reached for this animal model. Neuronal degeneration, although to a limited extent, was always co-localized with SARS-CoV-2 infection.

## Supplemental Material

Supplemental Material, sj-pdf-1-vet-10.1177_03009858211066841 - Chronological brain lesions after SARS-CoV-2 infection in hACE2-transgenic mice Click here for additional data file. Supplemental Material, sj-pdf-1-vet-10.1177_03009858211066841 for Chronological brain lesions after SARS-CoV-2 infection in hACE2-transgenic mice by Enric Vidal, Carlos López-Figueroa, Jordi Rodon, Mónica Pérez, Marco Brustolin, Guillermo Cantero, Víctor Guallar, Nuria Izquierdo-Useros, Jorge Carrillo, Julià Blanco, Bonaventura Clotet, Júlia Vergara-Alert and Joaquim Segalés in Veterinary Pathology

Supplemental Material, sj-xlsx-1-vet-10.1177_03009858211066841 - Chronological brain lesions after SARS-CoV-2 infection in hACE2-transgenic mice Click here for additional data file. Supplemental Material, sj-xlsx-1-vet-10.1177_03009858211066841 for Chronological brain lesions after SARS-CoV-2 infection in hACE2-transgenic mice by Enric Vidal, Carlos López-Figueroa, Jordi Rodon, Mónica Pérez, Marco Brustolin, Guillermo Cantero, Víctor Guallar, Nuria Izquierdo-Useros, Jorge Carrillo, Julià Blanco, Bonaventura Clotet, Júlia Vergara-Alert and Joaquim Segalés in Veterinary Pathology
