# Supplementary material for: Chronological brain lesions after SARS-CoV-2 infection in hACE2-transgenic mice
Source: Vet Pathol. 2021 Dec 27;59(4):613–26. doi: 10.1177/03009858211066841 (PMC9207990; doi:10.1177/03009858211066841)
Supplement: Supplemental Material, sj-pdf-1-vet-10.1177_03009858211066841 - Chronological brain lesions after SARS-CoV-2 infection in hACE2-transgenic mice [file sj-pdf-1-vet-10.1177_03009858211066841.pdf]

Supplemental Table S1: Immunohistochemical procedure details.

Primary Antibodies	Commercial reference	Dilution of primary antibody	Antigen retrieval and other treatments	Incubation of primary antibody	Visualization system
**SARS-CoV-2 NP** rabbit monoclonal	Sino Biological (40143-R019)	1:15000	Peroxidase block (Methanol 3% H_2O_2 30 min RT)<br><br>HIER Citrat Buffer pH6 (Dako Target Retrieval Solution) 98°C 20 min + 30 min RT	Overnight 4°C	EnVision®+ System linked to horseradish peroxidase (HRP, Agilent-Dako) and 3,3'-diaminobenzidine (DAB)
**GFAP** rabbit polyclonal	Dako (Z0334)	1:2000	Peroxidase block (3% H_2O_2 in H_2Od 35 min RT)<br><br>HIER citrate buffer, 0.01M pH6, 98ºC 20 min + 30 min RT	Overnight 4°C	EnVision®+ System linked to horseradish peroxidase (HRP, Agilent-Dako) and DAB.
**IBA1** goat polyclonal	ABCAM (ab5076)	1:300	Peroxidase block (3% peroxide hydrogen in H_2Od for 35 min RT)<br><br>HIER citrate buffer, 0.01M pH6, 98ºC 20min + 30 min RT	Overnight 4°C	Polyclonal rabbit anti-goat biotinylated secondary antibody (DAKO, 1:200) with the ABC Peroxidase Standard Staining Kit (Thermo Scientific™) and DAB
**CD3** rabbit monoclonal	Cell Signalling (clone D4V8L)	1:500	Peroxidase block (Methanol 3% H_2O_2 30 min TA)<br><br>HIER EDTA Buffer pH9 (Dako EnVision FLEX Target Retrieval Solution High pH) 98°C 20 min + 30 min RT	Overnight 4°C	EnVision®+ System linked to horseradish peroxidase (HRP, Agilent-Dako) and DAB.
**CD20** goat polyclonal	Santa Cruz Biotechnology (SC-7735)	1:250	Alkaline Phosphatase block (Bloxall Blocking Solution, Palex Medical) 15 min RT<br>HIER Citrat Buffer pH6 (Dako Target Retrieval Solution) 98°C 20 min + 30 min RT	Overnight 4°C	ImmPRESS®-AP -alkaline phosphatase linked-horse anti goat IgG Polymer (Maraval Life Sciences -Vector Laboratories) and a red colored chromogen (ImmPACT Vector Red Substrate Kit Alkaline Phosphatase, Vector Laboratories).

Legend: RT: room temperature; HIER: Heat induced epitope retrieval, min: minutes;
